# Comparision of crosslinked hyaluronic acid vs. enamel matrix derivative for periodontal regeneration: an 18-month follow-up randomized clinical trial

**DOI:** 10.1007/s00784-025-06278-5

**Published:** 2025-03-19

**Authors:** Manuel Rodríguez-A, Jose María Montiel-Company, Francisco Alpiste-Illueca, Lucía Rodríguez-A, Vanessa Paredes-Gallardo, Andrés López-Roldán

**Affiliations:** Department of Stomatology, Faculty of Medicine and Odontology, University of Valencia, Valencia, Spain

**Keywords:** Hyaluronic acid, Enamel matrix derivative, Periodontal regeneration, Randomized controlled trial, Radiographic image interpretation

## Abstract

**Aim:**

To compare the effects of 1.8% hyaluronic acid (HA) and enamel matrix derivative (EMD) on periodontal regeneration in patients with periodontal bone defects, using clinical and radiographic parameters as outcome measures.

**Materials and methods:**

We included 53 patients with 53 intrabony defects in this study who were randomly assigned to either the HA (test) or EMD (control) groups. Clinical and radiographic parameters were evaluated at 6, 12, and 18 months after the surgery.

**Results:**

Clinical measurements at 6, 12, and 18 months after surgery demonstrated significant improvements in probing depth (PD), clinical attachment level (CAL), recession (REC), and bleeding on probing for both groups compared with baseline (*p* < 0.001). The EMD group exhibited the highest CAL gain of 2–3 mm at 6 months, observed in 14 of 26 (53.8%) defect sites. Conversely, the HA group demonstrated a CAL gain ≥ 4 mm at 18 months, observed in 13 of 27 (48.1%) defect sites. Radiographic assessments at 6, 12, and 18 months demonstrated significant improvements from baseline for both groups (*p* < 0.001).

**Conclusion:**

We found significant clinical and radiographic benefits of HA and EMD at 18 months, with some limitations in effectiveness for specific intraosseous defects.

**Clinical relevance:**

This study demonstrated that hyaluronic acid (HA), combined with minimally invasive techniques, enhances periodontal regeneration by improving PPD reduction, CAL gain, and radiographic bone filling, with cost-effectiveness, application, and bioavailability surpassing that of other biomaterials. Based on these results, HA can be considered a viable alternative to EMD in indicated cases.

**Clinical trial registration number:**

clinicalTrial.gov - NCT04274244.

**Supplementary Information:**

The online version contains supplementary material available at 10.1007/s00784-025-06278-5.

## Introduction

The bone loss patterns in periodontitis, often associated with intrabony defects, are categorized based on the number of walls involved [1]. Treating these lesions is a priority, with regenerative surgery offering superior outcomes and prognosis compared to open flap debridement [2]. Current evidence supports the use of enamel matrix derivative (EMD) with guided tissue regeneration techniques, avoiding bone grafts and employing papillary preservation approaches [3, 4]. However, these procedures are technically demanding, with membrane exposure being a common complication, potentially reducing efficacy and requiring frequent follow-ups and additional treatments [5]. EMD plays a key role in promoting cementogenesis and the regeneration of the periodontal ligament is an alternative to membranes in certain cases but demands precision to prevent blood contamination, which impairs periodontal ligament cell adhesion and proliferation [6]. Advancing materials for periodontal regeneration remains essential to improve clinical outcomes and minimize challenges.

Hyaluronic acid (HA) is a promising biomaterial for periodontal regeneration due to its unique properties [7]. Its hygroscopic and viscoelastic nature allows it to retain water, transport metabolites, and maintain tissue structure, adapting through interactions with cells and matrix components [8]. HA has multiple beneficial properties including its being antimicrobial [9], osteoinductive [10] thus aiding periodontal healing [11]. It reduces gingival epithelium proliferation [12, 13] and promotes periodontal ligament cell adhesion and proliferation via CD44 receptor activation [14, 15]. Its effects depend on molecular weight and concentration [16], which are specific to each cell and influenced by the receptor expressed [12]. Its properties, including inflammation control, granulation, tissue remodeling, and clot stabilization, make HA a valuable material for periodontal regeneration [17].

Several clinical studies have evaluated the effect of HA alone or in combination with other biomaterials [18–20]. However, only one study, which had a small sample size [21], short follow-up period, and lacked radiographic evidence [22], has directly compared the effects of HA and EMD on intrabony defects. We hypothesized that HA exhibited similar potential for periodontal regeneration compared with that of EMD due to its well-documented bioactive properties that promote tissue regeneration. Therefore, this randomized controlled clinical trial aimed to compare the effects of 1.8% HA and EMD on periodontal regeneration following bone defects, utilizing both clinical and radiographic assessments.

## Materials and methods

### Experimental design

**Fig. 1:**
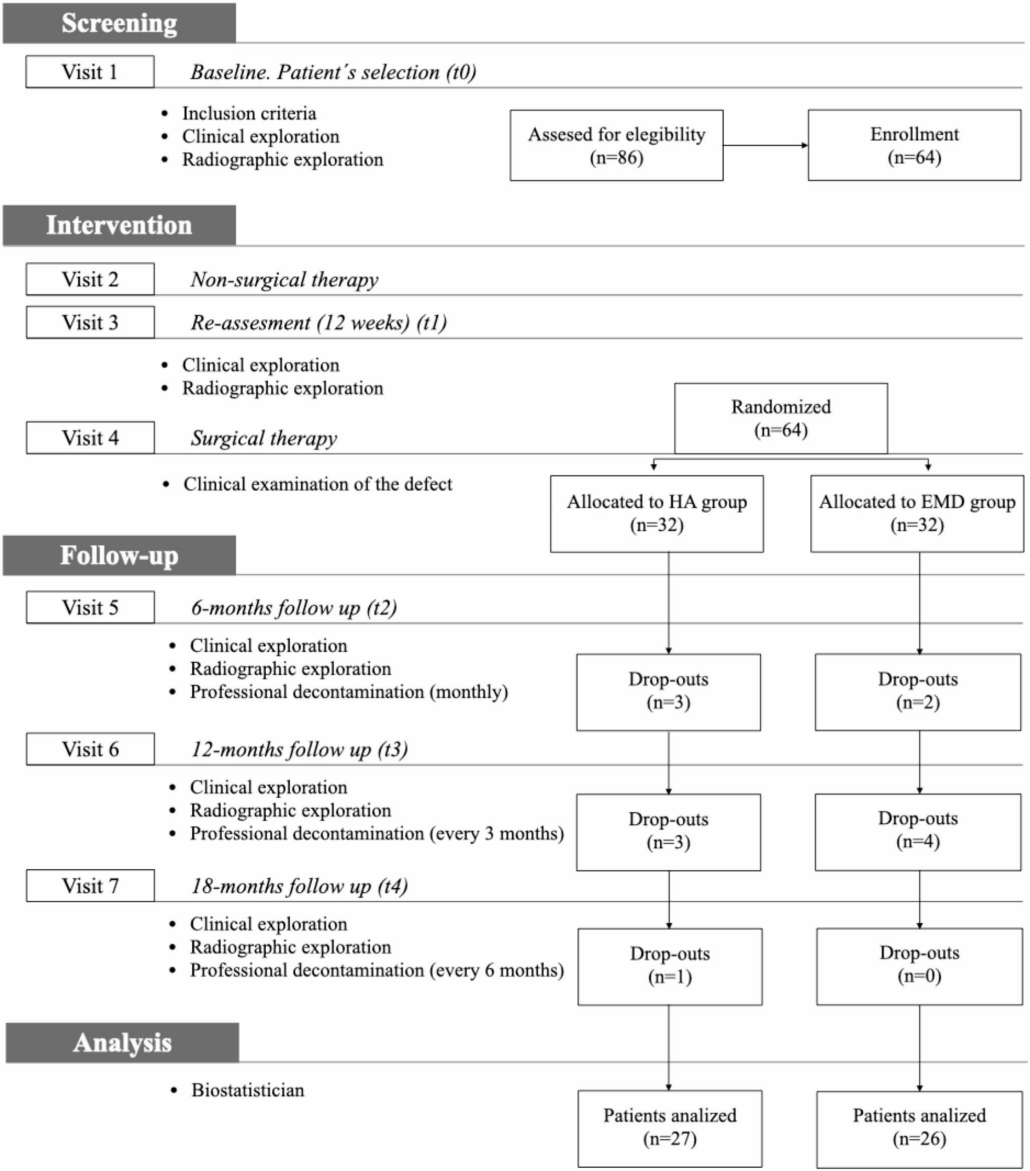
CONSORT flow chart

This randomized controlled clinical trial, employing a parallel design, included 53 patients (24 women and 29 men) aged between 35 and 60 years diagnosed with periodontitis stage III or IV, or grade A or B [23]. The efficacy of HA (Hyadent BG^®^; BioScience GmbH, Dümmer, Germany) and EMD (Emdogain^®^; Institut Straumann AG, Basel, Switzerland) in facilitating periodontal regeneration was compared across 53 intrabony defects, with 1 defect treated per patient. The study design and patient selection criteria are illustrated in Fig. 1.

The study was conducted in accordance with the principles of the Helsinki Declaration of 1975, as revised in 2013. The study protocol was reviewed and approved by the ethical committee of the University of Valencia, Spain (register code 1025213), and informed consent was obtained from all participants. The protocol adhered to the current standards of clinical research (CONSORT guidelines) (ClinicalTrial.gov - NCT04274244).

### Inclusion criteria

Patients were consecutively enrolled based on the following inclusion criteria:

- Absence of systemic diseases.
- Non-smokers or smokers consuming < 10 cigarettes/day.
- Good oral hygiene following non-surgical periodontal therapy (full-mouth plaque and bleeding scores ≤ 10%).
- Compliance with the maintenance program.
- Presence of a single intrabony defect with a probing depth (PD) ≥ 6 mm and a defect component ≥ 3 mm, confirmed by radiographs and intrasurgical examination.

Smoking habit was monitored throughout the study to assess the effect of tobacco on periodontal regeneration. All participants who were smokers received clinical support and motivation to quit or reduce their tobacco consumption.

### Clinical measurements

The following clinical parameters were assessed at baseline (t0), 3 months after non-surgical therapy (t1) and 6 (t2), 12 (t3), and 18 (t4) months after the surgical procedure using the same type of periodontal probe (PCP-UNC 15; Hu-Friedy Manufacturing, Chicago, IL, USA). The primary outcome was the clinical attachment level (CAL). Secondary outcomes included: [1] probing pocket depth [2], bleeding on probing [3], gingival recession (REC) [4], full-mouth bleeding score [5], full-mouth plaque score, and [6] tooth mobility [24].

### Radiographic measurements

Radiographs were obtained at t0–t4. The parallel technique (Rinn collimator, Dentsply/Rinn) and an intraoral X-ray machine using the Durr dental system (VistaScan, Dürr Dental AG, Germany) with digital phosphor plates were used to obtain reproducible radiographic images throughout the treatment. The following variables were recorded (Appendix Fig. 1) [25]: [1] cemento-enamel junction– bottom defect (CEJ-BD); [2] cemento-enamel junction– bottom crest (CEJ-BC); [3] INFRA (CEJ-BD–CEJ-BC); [4] wall component (x-WALL); [5] angle of intrabony defect; [6] area of regeneration; [7] 100% area defect; and [8] percentage defect filling.

The radiographic technique was standardized in areas with bone defects to assess their evolution and ensure consistency. Bite blocks were used to hold the digital system sensor. Previous impressions were obtained at baseline and saved for subsequent radiographic evaluations. Radiographic measurements taken at baseline and during surgery after debridement, were analyzed with Dental Image Analyzer software [26].

### Intra-examiner reproducibility

Five patients with 10 teeth each (PDs > 6 mm on at least one aspect) were used to calibrate the examiner. Evaluations were 48 h apart, with calibration deemed acceptable if baseline and follow-up measurements matched > 90% at the millimeter level. The examiner was blinded to the surgical procedure.

### Sample size

To conclude the non-inferiority of HA compared to EMD, with a non-inferiority margin of 30%, a standard deviation of 1.3 mm, a 95% confidence level, and 80% power, a sample size calculation determined that 23 participants per group were required. This corresponds to a total of 46 patients, assuming equal group sizes, to detect a standardized effect size of 0.5 in the difference of means for the primary outcome (CAL) under a two-sided hypothesis.

### Randomization and blinding

During surgery, the defects were randomly assigned to the HA and EMD groups. In each case, the surgeon was informed of the assigned treatment option after flap elevation and defect debridement. Randomization was performed using STATA (StataCorp LLC, College Station, TX, USA) and an envelope at the time of the surgery. The researchers who performed the measurements differed from those who performed the surgery, ensuring that both the examiner and the statistician remained blinded throughout the study. Similarly, the examiners underwent reproducibility tests to ensure reliability in image analysis.

### Pre-surgical phase

Each patient received oral hygiene instructions and non-surgical periodontal treatment (NSPT) for primary periodontitis control from June 2019 to December 2021. After 12 weeks of NSPT, clinical measurements were used to determine inclusion in the surgical phase, dismissing patients with spontaneous clinical and radiographic periodontal regeneration.

### Surgical procedure

**Fig. 2:**
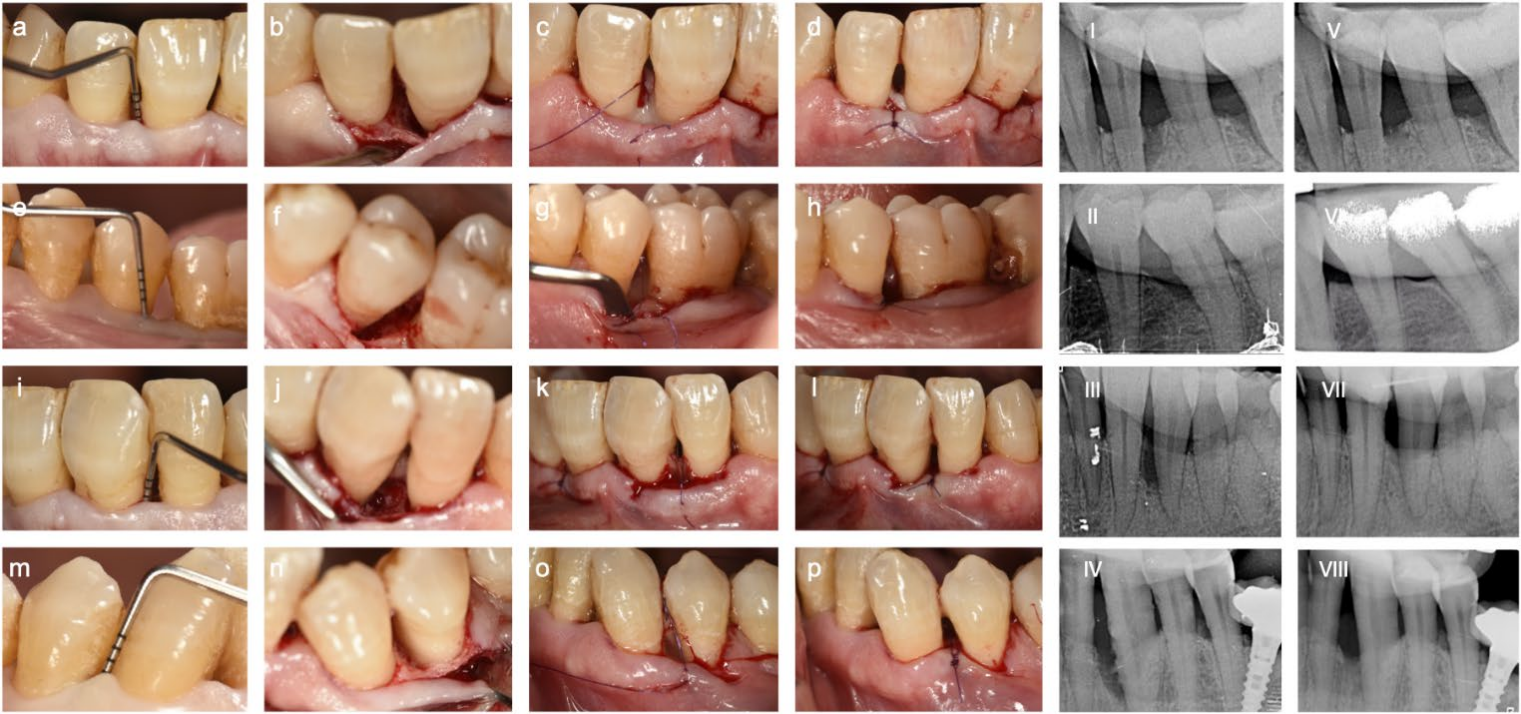
Representative bone defects treated by control (**a-h**) and test (**i-p**) therapies. Control group cases (EMD): (**a-e**) Baseline clinical view and pre-surgical probing, (**b-f**) Surgical view after flap elevation, **cg** Enamel matrix derivative (EMD) application, and (*d-h*) Suture closure. Test group cases (HA): (**i-m**) Baseline clinical view and pre-surgical probing, (**j-n**) Surgical view after flap elevation, (**k-o**) Enamel matrix derivative (EMD) application, and (**l-p**) Suture closure. Radiographs represents bone defects at baseline (I-IV) and 18 months (V-VI). The upper shadow represents the silicone bite block for standardized X-ray projection

All patients were treated at the Unit of Periodontology of the University of Valencia, Spain, by the same experienced surgeon (M.R.). The surgeries were performed between March 2020 and March 2022, following initial NSPT (Fig. 2). After administering local anesthesia, open flap surgical procedures were performed using the minimally invasive surgical technique (MIST) [27] or modified technique (M-MIST) [28], depending on the interdental space width and access [29]. All granulation tissue was removed from the defects, and the roots were thoroughly scaled and planed using hand and ultrasonic instruments. The defects were re-examined and measured during the surgery to confirm their anatomy, as outlined by the preoperative radiograph.

The defects were randomly assigned to treatment groups using envelopes after debridement. Both groups received a single loose internal modified mattress suture (6/0 or 7/0 e-PTFE Goretex, WL Gore & Associates). For the M-MIST + EMD group, 24% EDTA gel (PrefGel^®^, Straumann) conditioned the root surface to remove the smear layer [30], followed by EMD (Emdogain^®^, Straumann) application on the root and inside the defect to prevent contamination with blood. In the M-MIST + HA group, HA was used instead of EMD. Sutures were tightened for primary closure, with additional sutures as needed. All procedures were performed with 4× magnification and microsurgical instruments.

### Post-surgical instruction and infection control

A protocol for controlling bacterial contamination included amoxicillin (1.5 g/day for 1 week), 0.12% chlorhexidine rinses twice daily, and weekly prophylaxis. Patients avoided brushing, flossing, and chewing in the treated area for 3–4 weeks, resuming full oral hygiene afterward. A 3-month recall system was implemented for 18 months post-surgery. Supportive periodontal therapy included professional cleanings and clinical/radiographic evaluations every 3–6 months based on individual needs. During the 18-month follow-up, the treated site was decontaminated with mechanical instrumentation. Follow-up lasted from September 2020 to September 2023.

### Statistical analyses

Data from 53 defects in 53 patients were analyzed (IBM SPSS Statistics software). The normality of variable distribution was assessed using the Shapiro–Wilk test. Inferential analysis included Pearson and Spearman correlations for linear and non-linear associations, an independent samples *t-*test for normally distributed parameters between groups, and the Mann–Whitney test for non-normally distributed ordinal or continuous parameters. The significance level was set at 0.05, and the power of the study was calculated at 0.95 to detect a significant difference of 1 mm between groups.

## Results

We screened 86 patients, and after accounting for potential drop-outs, fifty-three participants completed the study, as shown in Fig. 1. The study population comprised 28 women and 25 men (each had 1 intrabony defect treated), aged 35–55 years. In the test group, 27 defects (13, 9, and 5 with 3-, 2-, and 1-wall defects, respectively) were treated. In the control group, 26 defects (10, 11, and 5 with 3-, 2-, and 1-wall defects, respectively) were treated. No significant differences were detected in the type and morphology of the analyzed defects, and no relevant complications were observed during the study period.

### Baseline measurements

The baseline characteristics between the two groups did not significantly differ (*p* < 0.05; Table 1).

**Table 1:** Baseline clinical and radiographic parameters by group

Demographic data	HA (*n* = 27)	EMD (*n* = 26)	*p*-value
*Age*	46.54 ± 9.32	44.23 ± 8.81	0.92^a^
*Gender (female/male)*	15/12	13/13	0.81^a^
*Smokers*	12	12	-
*Clinical parameters*			
CAL (mm)	9.11 ± 1.67	8.93 ± 1.18	0.91^b^
PPD (mm)	7.56 ± 1.54	7.37 ± 1.02	0.95^b^
REC (mm)	1.55 ± 1.06	1.56 ± 0.86	0.82^b^
BOP	0.89 ± 0.7	0.63 ± 1.1	0.66^a^
FMPS (%)	50.1 ± 13.2	49.4 ± 15.7	0.89^a^
FMBS (%)	40.13 ± 9.45	36.87 ± 12.13	0.72^a^
*Radiographic parameters*			
INFRA	5.8 ± 1.4	5.9 ± 1.1	0.81^b^
CEJ-BD	10.9 ± 1.5	11.6 ± 1.5	0.86^b^
CEJ-BC	5.2 ± 1.1	5.9 ± 0.9	0.77^b^
Defect angle	28.2 ± 8.0	27.8 ± 7.8	0.90^b^

^a^ Wilcoxon’s test

^b^ Wilcoxon-Mann–Whitney test

*HA* hyaluronic acid; *EMD* enamel matrix derivatives; *CAL* clinical attachment level; *PPD* probing pocket depth; *REC* recession; *BOP* bleeding on probing; *FMPS* full-mouth plaque score; *FMBS* full-mouth bleeding score; INFRA infraosseous defect; CEJ-BD cemento-enamel junction to bottom defect; CEJ-BC cemento-enamel junction to bone crest

Level of significance *p* < 0.05

### Clinical measurements at 3, 6, 12, and 18 months

The clinical measurements at 12 weeks were comparable in both groups, with probing pocket depth values of 6.77 ± 0.63 and 7.04 ± 0.87 mm for the HA and EMD groups, respectively. Additionally, the CAL values were 9.47 ± 1.23 and 9.70 ± 1.10 mm for the AH and EMD groups, respectively. However, these differences were not significant compared with those of the baseline.

Clinical measurements at 6, 12, and 18 months post-surgery are summarized in Table 2. Both groups exhibited significant improvements in PD, CAL, REC, and bleeding on probing compared with those of their baseline measurements (*p* < 0.001). The mean PD reduction was more pronounced in the EMD group at 6, 12, and 18 months (3.79 ± 1.35, 4.42 ± 1.64, and 4.38 ± 1.50 mm, respectively) than in the HA group (3.56 ± 1.72, 3.80 ± 1.39, and 3.96 ± 1.41 mm, respectively. The CAL gains at 6, 12, and 18 months were comparable between the EMD (3.05 ± 1.61, 3.58 ± 1.92, and 3.50 ± 1.81 mm, respectively) and AH (3.19 ± 1.75, 3.36 ± 1.55, and 3.43 ± 1.62 mm, respectively) groups. At 6, 12, and 18 months, the REC increase was lower in the AH group (0.40 ± 0.42, 0.43 ± 0.44, and 0.53 ± 0.53 mm, respectively) than in the EMD group (0.74 ± 0.54, 0.84 ± 0.60, and 0.88 ± 0.64 mm, respectively).

**Table 2:** Clinical parameters of the AH and EMD groups at 6 (t2), 12 (t3), and 18 (T4) months

Parameter	6 months (t2)			12 months (t3)			18 months (t4)		
	Mean ± SD	Median [IQR]	*p*-value	Mean ± SD	Median	*p*-value	Mean ± SD	Median	*p*-value
***CAL (mm)***									
HA	6.33 ± 1.97	5.50 [3.0]	< 0.001^a^***	6.16 ± 1.76	6.00 [2.4]	< 0.001^a^***	6.09 ± 1.91	5.50[3.0]	< 0.001^a^***
EMD	6.39 ± 1.41	6.00 [2.3]	< 0.001^a^***	5.85 ± 1.45	5.75 [1.6]	< 0.001^a^***	5.93 ± 1.23	6.00[2.0]	< 0.001^a^***
HA vs. EMD	*r* = 0.09		0.496^b^	*r* = 0.07		0.616^b^	*r* = 0.04		0.788^b^
***CAL gain (mm)***									
HA	3.19 ± 1.75	3.00 [3.0]	< 0.001^c^***	3.36 ± 1.55	3.50 [2.0]	< 0.001^c^***	3.43 ± 1.62	3.50 [2.5]	< 0.001^c^***
EMD	3.05 ± 1.61	3.00 [2.4]	< 0.001^c^***	3.58 ± 1.92	3.75 [2.5]	< 0.001^c^***	3.50 ± 1.81	3.50 [2.3]	< 0.001^c^***
HA vs. EMD	d = 0.02		0.759^e^	d = 0.13		0.650^e^	d = 0.04		0.882^e^
***PPD (mm)***									
HA	3.63 ± 1.36	3.00 [2.0]	< 0.001^a^***	3.39 ± 1.0	3.00 [0.50]	< 0.001^a^***	3.22 ± 1.18	3.00 [0.50]	< 0.001^a^***
EMD	3.69 ± 1.92	4.00 [1.0]	< 0.001^a^***	3.06 ± 0.85	3.00 [1.50]	< 0.001^a^***	3.10 ± 1.60	3.00 [0]	< 0.001^a^***
HA vs. EMD	*r* = 0.11		0,411^b^	*r* = 0.16		0,233^b^	*r* = 0.08		0,556^b^
***PD reduction (mm)***									
HA	3.56 ± 1.72	3.50 [3.0]	< 0.001^c^***	3.80 ± 1.39	4.00 [1.50]	< 0.001^c^***	3.96 ± 1.41	4.00 [2.0]	< 0.001^c^***
EMD	3.79 ± 1.35	4.00 [1.50]	< 0.001^c^***	4.42 ± 1.64	4.25 [2.50]	< 0.001^c^***	4.38 ± 1.50	4.25 [2.50]	< 0.001^c^***
HA vs. EMD	d = 0.15		0.586^e^	d = 0.41		0.138^e^	d = 0.29		0.296^e^
***REC (mm)***									
HA	2.74 ± 1.16	3.00 [1.50]	< 0.001^a^***	2.77 ± 1.18	2.50 [1.50]	< 0.001^a^***	2.86 ± 1.26	3.00 [1.50]	< 0.001^a^***
EMD	2.70 ± 1.89	2.50 [1.30]	< 0.001^a^***	2.80 ± 1.88	2.75 [1.60]	< 0.001^a^***	2.83 ± 0.95	2.75 [1.60]	< 0.001^a^***
HA vs. EMD	*r* = 0.02		0,871^b^	*r* = 0.04		0,780^b^	*r* = 0.01		0,978^b^
***REC increase (mm)***									
HA	0.40 ± 0.42	0.50 [0.50]	0.052^b^	0.43 ± 0.44	0.50 [0.50]	0.122^a^	0.53 ± 0.53	0.50 [1.0]	0.052^a^
EMD	0.74 ± 0.54	0.55 [0.50]	0.002^a**^	0.84 ± 0.60	0.80 [0.50]	0.001^a**^	0.88 ± 0.64	0.80 [0.90]	0.001^a**^
HA vs. EMD	*r* = 0.32		0.019^b^*	*r* = 0.36		0.009^b^**	*r* = 0.28		0.041^b^*
***BOP***									
HA	0.27 ± 0.39	0.00 [1.00]	< 0.001^a^***	0.20 ± 0.36	0.00 [1.00]	< 0.001^a^***	0.19 ± 0.39	0.00 [1.00]	< 0.001^a^***
EMD	0.32 ± 0.41	0.00 [1.00]	< 0.001^a^***	0.29 ± 0.31	0.00 [1.00]	< 0.001^a^***	0.3 ± 0.45	0.00 [1.00]	< 0.001^a^***
HA vs. EMD	*r* = 0.14		0.309^b^	*r* = 0.03		0.843^b^	*r* = 0.01		0.941^b^
***FMPS***									
HA	9.0 ± 1.1	0.50 [0.50]	< 0.001^a^***	8.4 ± 1.1	0.00 [1.00]	0,411 ^a^	8.9 ± 3.3	0.00 [1.00]	0,449 ^a^
EMD	8.6 ± 0,9	0.50 [0.50]	< 0.001^a^***	8.4 ± 1.0	0.00 [1.00]	0,721 ^a^	9.1 ± 2.8	0.00 [1.00]	0,701 ^a^
HA vs. EMD	*r* = 0.17		0,531 ^b^	*r* = 0.03		0,472 ^b^	*r* = 0.02		0,819 ^b^
***FMBS***									
HA	5.3 ± 2.3	0.00 [1.00]	< 0.001^a^***	2.8 ± 0,9	0.00 [1.00]	0,372 ^a^	3.3 ± 3.0	0.00 [1.00]	0,679 ^a^
EMD	6.1 ± 1.4	0.00 [1.00]	< 0.001^a^***	2.0 ± 1.5	0.00 [1.00]	0,581 ^a^	2.5 ± 3.8	0.00 [1.00]	0,885 ^a^
HA vs. EMD	*r* = 0.15		0,210 ^b^	*r* = 0.29		0,037 ^b^	*r* = 0.03		0,491 ^b^

^a^ Wilcoxon’s test

^b^ Wilcoxon-Mann–Whitney test

^c^ Paired t-test

^e^ Two-sample t-test

*HA* hyaluronic acid; *EMD* enamel matrix derivatives; *SD* standard deviation; *IQR* interquartile range; *CAL* clinical attachment level; P*PD* probing pocket depth; *REC* recession; *BOP* bleeding on probing

*p < 0.05 indicates significant differences

**p < 0.01 indicates significant differences

***p < 0.001 indicates significant differences

r = z _U−MW_ / $\:\sqrt{n}$ indicates effect size for Mann-Whitney’s test (*r* < 0.1 means small, *r* ≈ 0.3 means medium, *r* > 0.5 means large)

d is Cohen’s d effect size for mean differences (d = 0.2 means small, d = 0.5 means medium, d = 0.8 means large)

Measurements between the two groups did not significantly differ (*p* < 0.05). Comparison of clinical outcomes between 6 and 18 months indicated further improvements in PD reduction and CAL gain in the AH (3.56 ± 1.72 and 3.19 ± 1.75 mm, respectively) and EMD (3.79 ± 1.35 and 3.05 ± 1.61 mm, respectively) during the first 6 months. In the test group, the number of defect walls (x-WALL) was significantly correlated with numerous clinical variables, mostly at t2 and t3. The data for PD, REC, and CAL, especially CAL gain, showed significant improvements (*p* < 0.001) with an increasing number of walls (Appendix Fig. 2). The control group also experienced improvement with an increasing number of walls, although it was not significant (*p* > 0.05). Smoking cessation considerably decreased, from 44.4 to 46.2% at baseline to 22.2 and 30.8% at 18 months post-regeneration surgery in the HA and EMD groups, respectively. In both the test and control groups, CAL gain, PD reduction, and REC increase showed statistically significant improvements, with the most pronounced effects observed at T1 and T3.

### Frequency distribution

The frequency distribution of clinical and radiographic parameters is presented in appendix Fig. 3.

#### CAL gain

The highest CAL gain was observed at 6 months: 2–3 mm at 14 sites (53.8%) in the EMD group and ≥ 4 mm at 13 sites (48.1%) in the HA group.

#### PD reduction

The greatest PD reduction was within the range of 3–4 mm for both groups. These were observed at 6 months in the EMD group (17 sites [65.4%]) and 12 months in the HA group (17 sites [62.9%]). Notably, the HA group demonstrated a greater reduction in the 0–2-mm range, observed at 7 sites (25.9%) at 6 months.

#### REC increase

The greatest increase in REC was observed at 6 months in the EMD group, showing a 1-mm increase at 11 sites (42.1%). During the same period, the HA group exhibited a 1-mm REC increase at 5 sites (18%), showing a significant difference (*p* < 0.05).

#### Defect filling

The highest defect filling was 66.6% at 18 sites in the HA group and 72.2% at 19 sites in the EMD group, both observed at 6 months. No significant between-group differences were found at 12 or 18 months.

#### Radiographic regeneration area

At 6 months, the radiographic regeneration areas were 11.5 and 13.2 mm² in the HA and EMD groups, respectively, both increased compared with that at baseline. These areas continued to grow significantly until 18 months, measuring 18.1 and 18.3 mm² in the HA and EMD groups, respectively.

### Radiographic measurements at 6, 12, and 18 months

In both groups, the radiographic measurements at 6, 12, and 18 months demonstrated significant improvements compared with those at baseline (*p* < 0.001 and *p* < 0.01 for AH and EMD groups, respectively) (Table 3). However, no significant differences between groups were observed (*p* > 0.05).

**Table 3:** Radiographic parameters of AH and EMD group at 6 (t2), 12 (t3), and 18 (t4) months

6 months (t2)				12 months (t3)			18 months (t4)		
Parameter	Mean ± SD	Median [IQR]	*p*-value	Mean ± SD	Median [IQR]	*p*-value	Mean ± SD	Median [IQR]	*p*-value
INFRA (mm)									
HA	2.1 ± 1.1	2.0 [0.5]	< 0.001^a^***	1,3 ± 1.5	1.1 [0.5]	0.031^a^*	1,2 ± 1.3	1.0 [0.5]	0.40^a^
EMD	2.0 ± 1.4	2.0 [0.5]	< 0.001^a^***	1.1 ± 1.3	1.0 [0.5]	0.047^a^*	1.0 ± 1.4	1.0 [0.5]	0.33^a^
HA vs. EMD	*r* = 0.04		0.815^b^	*r* = 0.07		0.652^b^	*r* = 0.10		0.515^b^
RX REG AREA (mm^2^)									
HA	10.9 ± 4.5	11.7 [8.3]	0.006^a^**	12.9 ± 5.2	13.4[10.1]	0.029^a^*	13.1 ± 5.3	13.6[10.2]	0.23^a^
EMD	10.7 ± 4.1	11.4 [5.0]	0.002^a^**	13.4 ± 4.6	13.3[6.0]	0.001^a^**	13.4 ± 4.7	13.3[12.5]	0.42^a^
HA vs. EMD	*r* = 0.011		0.934^b^	*r* = 0.05		0.735^b^	*r* = 0.04		0.762^b^
% DEFECT FILLING									
HA	66.6 ± 21.2	68.8[28.5]	< 0.001^a^***	78.7 ± 22.7	88.3[16.8]	0.001^a^**	79.5 ± 22.9	88.0[19.8]	0.32^a^
EMD	67.6 ± 20.9	71.5[19.8]	< 0.001^a^***	83.7 ± 20.2	88.9[13]	0.001^a^**	84.1 ± 21	90.6[14.3]	0.18^a^
HA vs. EMD	*r* = 0		1^b^	*r* = 0.12		0.374^b^	*r* = 0.13		0.346^b^

^a^ Wilcoxon’s test

^b^ Wilcoxon-Mann–Whitney test

*HA* hyaluronic acid; *EMD* enamel matrix derivatives; *SD* standard deviation; *IQR* interquartile range; RX REG AREA Radiographic regeneration area

*p < 0.05 indicates statistically significant differences

**p < 0.01 indicates statistically significant differences

***p < 0.001 indicates significant differences

r = z _U−MW_ / $\:\sqrt{n}$ indicates effect size for Mann-Whitney’s test (*r* < 0.1 means small, *r* ≈ 0.3 means medium, *r* > 0.5 means large)

At 6 months, the INFRA measurements corresponding to the radiographic intrabony depth demonstrated significant reductions of 2.1 ± 1.1 and 2 ± 1.4 mm for the AH and EMD groups, respectively (*p* < 0.001). Although no significant differences were found between groups, significant relationships were detected only between the radiographic regeneration area and x-WALLS for the test group (*p* < 0.01). This indicated that the regenerated area was greater with an increasing number of walls present in the defect (Appendix Fig. 4). However, the statistical relevance was lost at 12 and 18 months.

The area of regeneration and percentage defect filling measurements exhibited highly significant differences within both groups at 6 and 12 months (Table 3) but did not show significant differences between the groups. This was further supported by the CAL and radiographic regeneration area prediction models (Appendix Table 1 and Appendix Fig. 5).

## Discussion

This randomized clinical trial evaluated the effects of 1.8% HA (Hyadent BG^®^) and EMD (Emdogain^®^) on intrabony defects using clinical and radiographic periodontal parameters. After 18 months, no significant differences were observed between the two groups, except for the increase in REC, which was significantly higher in the EMD group.

To our knowledge, the only methodologically comparable study was conducted by Pilloni [21], who compared the effects of HA and EMD on intrabony defects and reported clinical outcomes similar to those of the present study. They reported higher but non-significant CAL gain and PD reduction in the EMD group. However, no significant differences were found between the groups for any clinical parameters, except for the increase in gingival REC, which was significantly greater in the EMD group. These results are in accordance with those observed in a recent study that also reported no statistically significant differences between groups regarding clinical measurements [22], however, this study presents significant limitations that affect the reliability of its findings, such as the use of OFD instead of minimally invasive techniques, a follow-up period limited to six months, and the lack of standardization in radiographic variables. Other studies examined and compared the effects of HA with those of open flap debridement [19, 31], membranes [20], HA combined with bone grafts [32], or its use as a vehicle for growth factors [33]. Clinical improvements and significant radiographic findings were noted for the HA group in some studies [32–34].

The only studies involving a radiographic assessment of bone filling was conducted by Sehdev [20] and Vela [22]; however, the use of non-standardized radiographs with varying projections affected the accuracy of their result analysis. In contrast, we used silicone bite blocks to ensure consistent repositioning of the X-ray plates, minimizing projection differences. Risk factors contributing to periodontal defects, such as developmental grooves, dental malposition, or mechanical trauma, can be addressed during the pre-surgical phase to promote spontaneous regeneration [3, 35]. To ensure the inclusion criteria were met, clinical and radiographic assessments were conducted at 12 weeks to confirm the defect’s suitability for the present study.

The CAL gain, PD reduction, and REC increase were similar when correlated with the x-WALLS, but both CAL gain and PD reduction for 1-wall defects were lower in the AH group. The sample size and reduced statistical power limited the strength of the conclusions when these parameters were isolated. However, the relationship between the number of walls and other variables was not significant in the EMD group. Conversely, smoking had a more pronounced effect when analyzed in relation to defect wall number. CAL gain, PD reduction, and REC increase were more significant with reduced tobacco consumption. The 80% smoking cessation rate among the participants in this study may have improved tissue and primary wound healing in the interdental spaces, although the cessation duration may have been insufficient to determine improvements in periodontal regeneration. A dose-dependent negative effect of tobacco consumption on periodontal regeneration has been reported, with smokers consuming > 10 cigarettes/day experiencing significantly lower CAL gain than non-smokers [36–38]. Other factors, such as a smaller radiographic angle of the intraosseous defect, were associated with greater radiographic regeneration area and defect filling, as narrower and deeper defects showed greater periodontal regeneration [39–41]. Among patients who completed the follow-up, CAL gain and PD reduction were most notable at 6 months and progressively decreased at 12 and 18 months.

HA characteristics such as molecular weight, crosslinking, and concentration vary across studies, highlighting the need for further research to fully understand their effects on periodontal regeneration. Zhao et al. [42] concluded that higher molecular weights (up to 2,630 kDa) were associated with reduced cell adhesion but increased cell proliferation. Conversely, higher HA concentrations (up to 2 mg/mL) were associated with increased adhesion and cell proliferation. Therefore, higher molecular weights and concentrations result in greater microRNA expression of typical osteogenic differentiation genes and increased formation of mineralized calcium nodules, indicating earlier, more rapid, and pronounced osteogenesis.

This study has limitations, as a true negative control was not included and defect measurements were limited to mm² radiographically, focusing only on the interproximal area rather than the full extent of combined defects. Volumetric (mm³) measurements would be more comprehensive but require invasive techniques with accuracy challenges. Larger studies are needed to assess wall number and biomaterial benefits fully.

## Conclusion

This trial showed significant clinical and radiographic benefits with both HA and EMD at 18 months. HA appears cost-effective, easy to use, and has fewer post-surgical complications, making it a viable option for intraosseous defect regeneration. Given the comparable outcomes between HA and EMD, hyaluronic acid—when formulated at the appropriate concentration and used in the indicated cases—may be considered a viable biomaterial for routine clinical practice.

## Electronic supplementary material

Below is the link to the electronic supplementary material.

Supplementary Material 1

## Data Availability

No datasets were generated or analysed during the current study.
