# Supplementary material for: Comparision of crosslinked hyaluronic acid vs. enamel matrix derivative for periodontal regeneration: an 18-month follow-up randomized clinical trial
Source: Clin Oral Investig. 2025 Mar 19;29(4):197. doi: 10.1007/s00784-025-06278-5 (PMC11922988; doi:10.1007/s00784-025-06278-5)
Supplement: Supplementary file 1 — Supplementary Material 1 [file 784_2025_6278_MOESM1_ESM.docx]

**Crosslinked Hyaluronic Acid vs Enamel Matrix for Periodontal Regeneration: Randomized Clinical Trial**

Rodríguez-A Manuel^1^, Montiel-Company Jose María^1^, Alpiste-Illueca Francisco^1^, Rodríguez-A Lucía^1^, Paredes- Gallardo Vanessa^1^, López-Roldán Andrés^1^

^1^Department of Stomatology, Faculty of Medicine and Odontology, University of Valencia, Valencia, Spain

**Corresponding Author:** Vanessa Paredes-Gallardo

vanessa.paredes@uv.es

**APPENDIX FIGURES AND TABLES**

***APPENDIX FIGURES***

***Appendix figure 1.*** *Reference points for evaluation of radiographic change. Variables recorded: (1) distance from the cemento-enamel junction (CEJ) to the bottom of the defect (BD; CEJ-BD), expressed to the nearest mm; (2) distance from the CEJ to the most coronal extension of the interdental bone crest (BC; CEJ-BC), expressed to the nearest mm; (3) INFRA (the intrabony component of the defect, calculated as [CEJ-BD–CEJ-BC]), expressed in mm; (4) wall component (x-WALL), representing the number of walls affected by the intrabony defect; (5) angle of intrabony defect, defined by the two lines representing the root surface and bone defect surface (A1-D1/B1-D1); (6) area of regeneration, referring to the measurement of the surface of regeneration compared with the radiograph at t1, measured at t2–t4, expressed in mm²; (7) 100% area defect, defined as the area of full regenerative capacity of the initial bone defect, corresponding to the area included in the infrabony defect between A1D1 and B1B; and (8) percentage defect filling, referring to the percentage of regeneration in tX compared with the 100% area defect.*


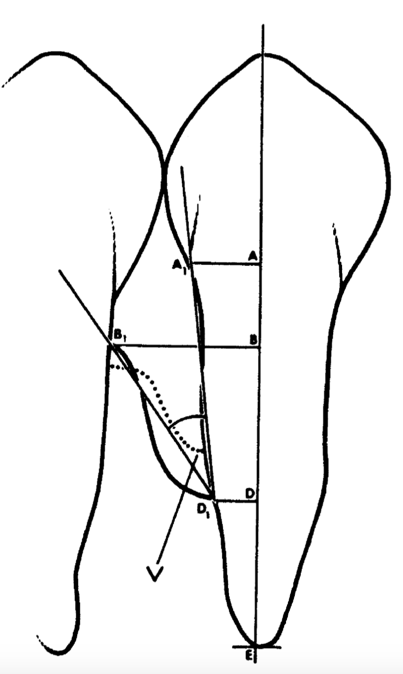


***Appendix figure 2.*** *Correlation between the number of bone defect walls and CAL gain at 6 (t2), 12 (t3) and 18 (t4) months in the test group.*


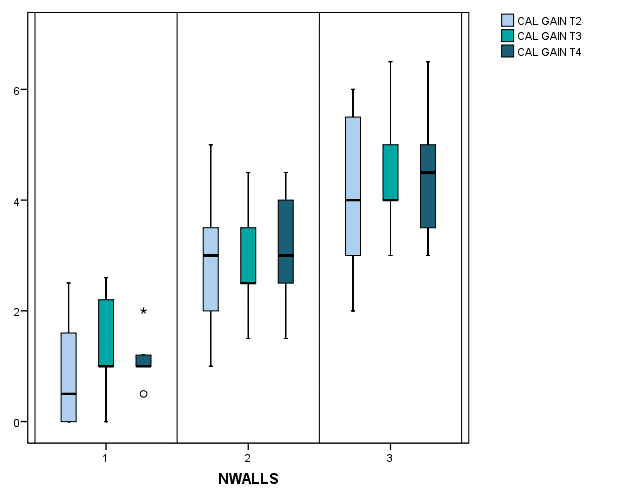


***Appendix figure 3.*** *Frequency distribution of clinical (a) and radiographic (b) parameters: CAL gain, PD reduction, and REC increase and % defect filling and radiographic regeneration area (mm^2^) at 6 (T2), 12 (T3), and 18 (T4) months.*


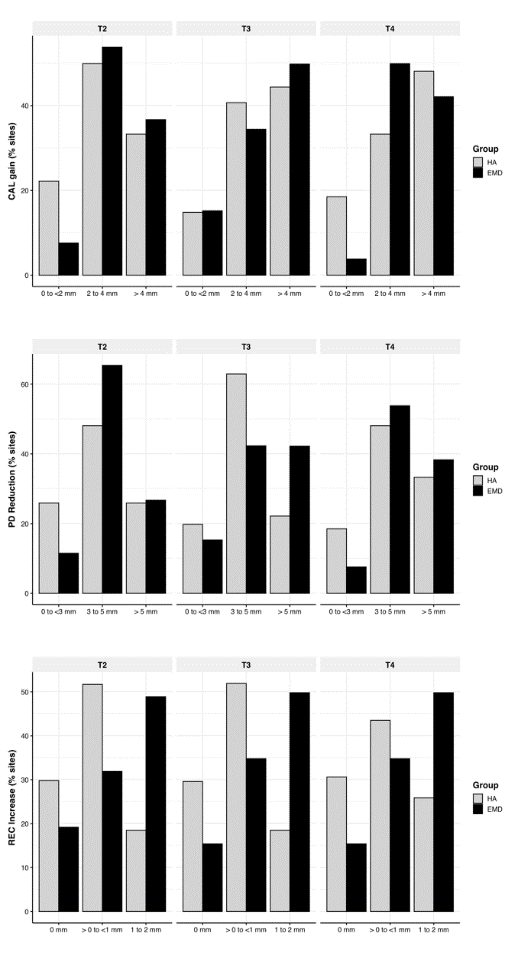

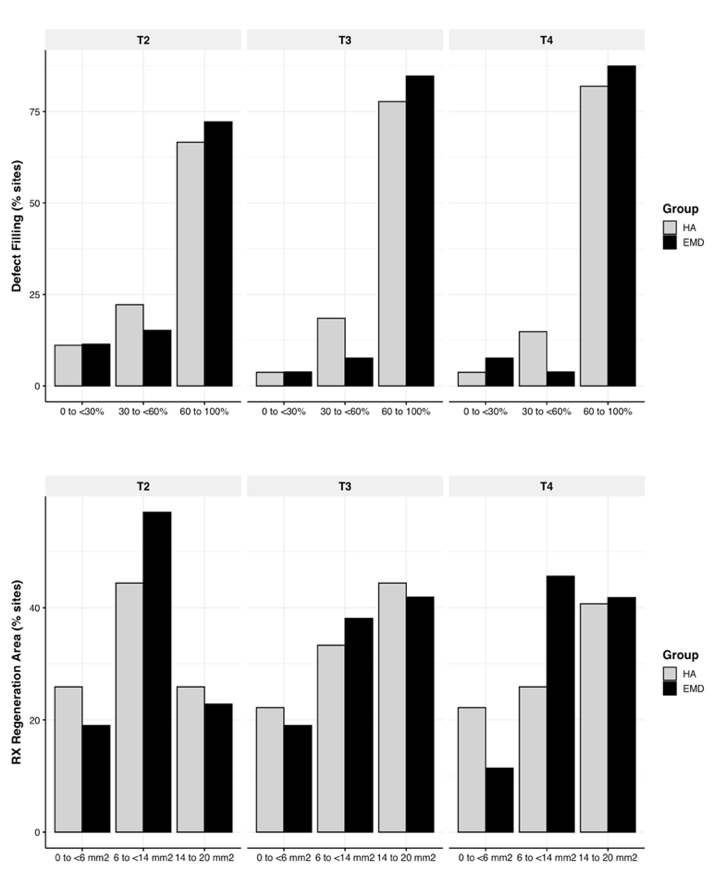


a.

b.

***Appendix figure 4.*** *Correlation between the number of walls of the bone defect and the radiographic regeneration area at 6 (t2), 12 (t3) and 18 (t4) months in the test and control group.*


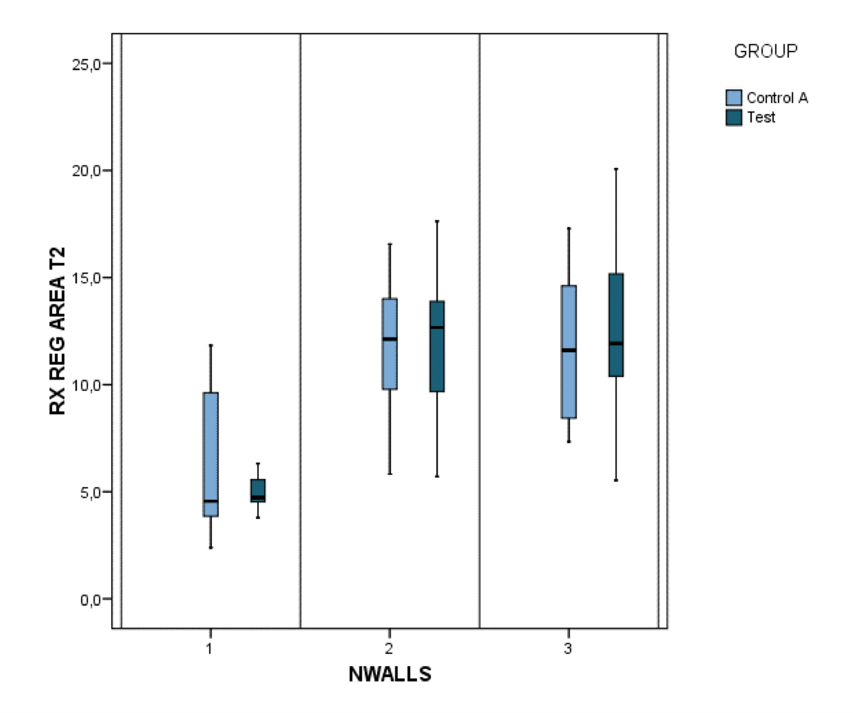


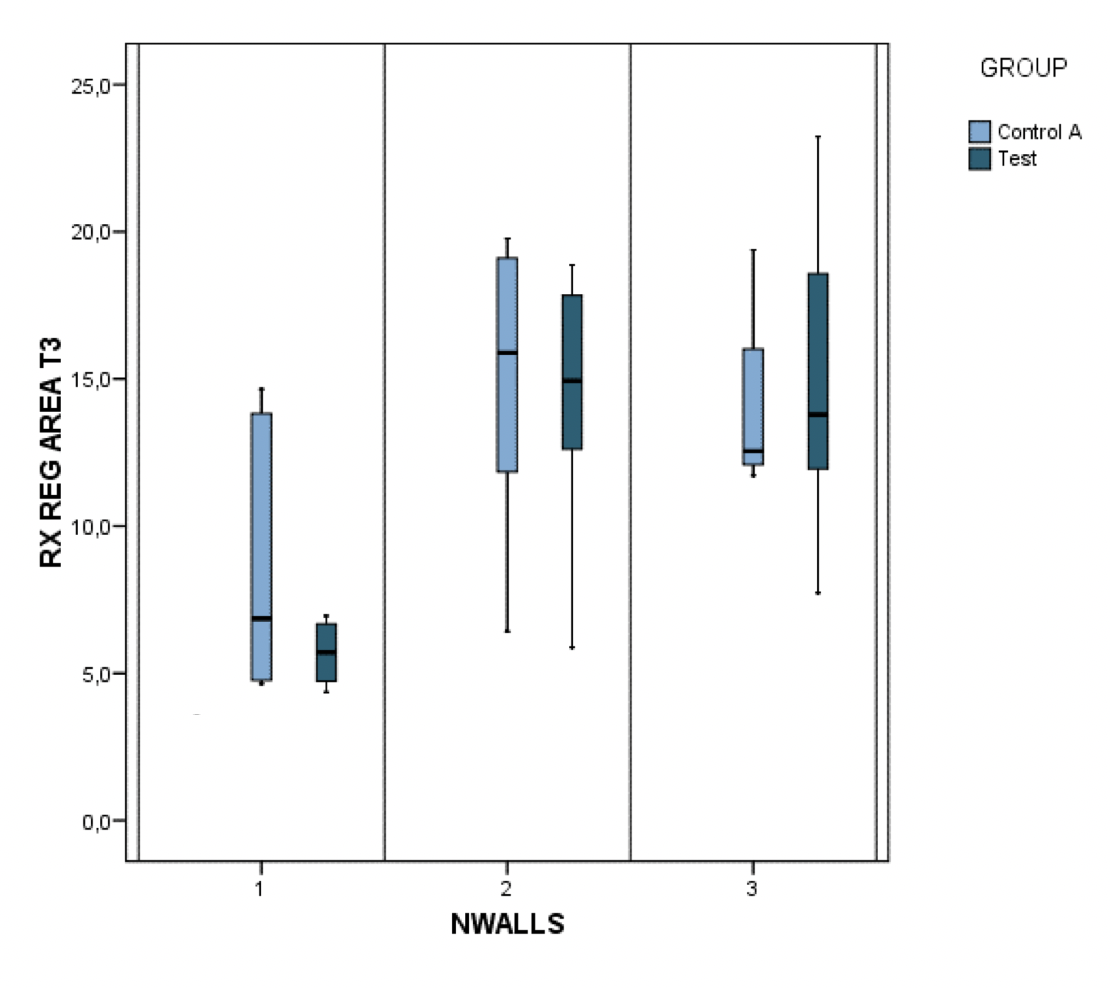


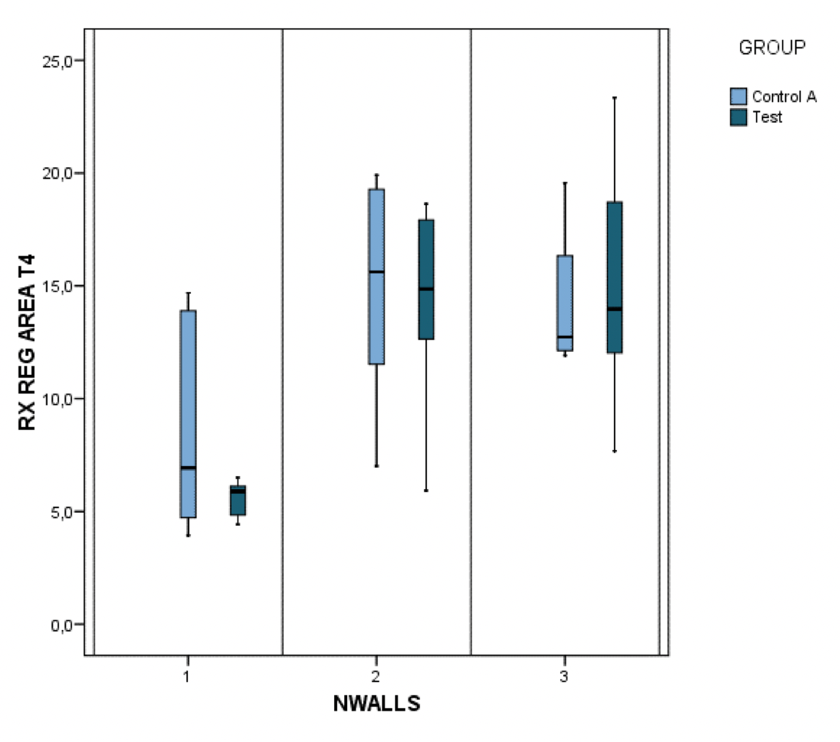


***Appendix* figure 5.** Correlation in a scatter diagram of the values of the prediction model for CAL (mm) with a linear correlation of Pearson R= 0.673 and radiographic regeneration area (mm^2^) with R=0.797.

***APPENDIX TABLE***

***Appendix table 1.*** *Predictive models of CAL (mm) and radiographic regeneration area (mm^2^). In the predictive model of CAL (R^2^=0.453), both the follow-up (for each month between 1 and 18 months the CAL decreases 0.18 mm) and the number of defect walls (2 or 3 initial defect walls predict a decrease of 2.06 mm) showed its significance. They did not show significant intragroup differences (HA or EMD) or smoking. In the predictive model of the radiographic regeneration area (R^2^=0.653), both the follow-up (for each month the defect area decreases 0.70 percentage units) and the number of defect walls (2 or 3 initial defect walls predict a decrease of 6.1 percentage units) showed its significance. Smoking, radiographic angle, CEJ-BC, or CEJ-BD did not show significant intergroup differences.*

|  |  | **Coefficient B non-standardized** | **Desv. error** | **Coefficient B standardized** | **p-value** |
| --- | --- | --- | --- | --- | --- |
| **CAL (mm)**  R=0.673; R^2^=0.453  p-valor <0.001 | ***Intercept*** | 10.249 | 0.344 |  | <0.001 |
|  | ***Follow-up (months)**** | -0.181 | 0.017 | -0.565 | <0.001 |
|  | ***2 or 3 walls**** | -2.063 | 0.284 | -0.376 | <0.001 |
|  | *Smoker* | -0.147 | 0.241 | -0.032 | 0.544 |
|  | *Hyaluronic acid* | 0.124 | 0.222 | 0.029 | 0.578 |
| **RX REG AREA (mm^2^)**  R=0.673; R^2^=0.453  p-valor <0.001 | ***Intercept*** | 15.250 | 2.739 |  | <0.001 |
|  | ***Follow up (months)**** | -0.697 | 0.043 | -0.698 | <0.001 |
|  | ***2 or 3 walls**** | -6.134 | 0.776 | -0.358 | <0.001 |
|  | *Radiographic angle baseline* | 0.042 | 0.038 | 0.049 | 0.273 |
|  | *Hyaluronic acid* | 0.616 | 0.612 | 0.046 | 0.315 |
|  | *CEJBD baseline* | 0.204 | 0.221 | 0.045 | 0.356 |
|  | *Smoker* | 0.423 | 0.650 | 0.030 | 0.516 |
|  | *CEJBC baseline* | -0.171 | 0.332 | -0.027 | 0.606 |
